# MicroRNA-451 is downregulated in the follicular fluid of women with endometriosis and influences mouse and human embryonic potential

**DOI:** 10.1186/s12958-019-0538-z

**Published:** 2019-11-19

**Authors:** Xiong Li, Wenbi Zhang, Jing Fu, Yan Xu, Ruihuan Gu, Ronggui Qu, Lu Li, Yijuan Sun, Xiaoxi Sun

**Affiliations:** 1 Shanghai Ji Ai Genetics & IVF Institute, Obstetrics and Gynecology Hospital, Fudan University, Shanghai, 200011 China; 2 Key Laboratory of Female Reproductive Endocrine Related Diseases, Obstetrics and Gynecology Hospital, Fudan University, Shanghai, 200011 China

**Keywords:** Follicular fluid, miR-451, Embryogenic potential, Endometriosis, Oocyte

## Abstract

**Background:**

Previous work demonstrated that there are numerous miRNAs in human follicular fluids, some of which are associated with reproductive diseases. In the current study, we sought to determine whether microRNAs (miRNAs) in the follicular fluid (FF) are differentially expressed between women with and without endometriosis and to uncover the association of miRNAs with the oocyte and embryonic development potential.

**Methods:**

FF was harvested from 30 women with endometriosis and 30 women without who underwent in vitro fertilization treatment at the University Hospital between February and December 2016. The FF samples were subjected to miRNA profiling and validation via quantitative reverse transcription polymerase chain reaction analysis. Mouse/human metaphase-I (MI) oocytes were harvested and micro-injected with an miR-451 inhibitor, and the effects of miR-451 knockdown on Wnt/WNT signalling genes were investigated.

**Results:**

Oocyte number, fertilization rate, and number of available embryos were decreased significantly in women with endometriosis relative to those without endometriosis. Hsa-miR-451 in FF was downregulated in endometriosis patients relative to control subjects (*P* < 0.01). Moreover, the proportions of mouse/human MI oocytes that developed into 2-pronuclei (2PN), 2-cell, 8–10-cell and blastocyst-stage embryos were affected by miR-451 knockdown in mouse/human oocytes. Components of the Wnt signalling pathway were aberrantly expressed in the mouse/human oocytes and embryos in the miR-451 inhibitor-injected group.

**Conclusions:**

miR-451 was downregulated in FF samples from endometriosis patients and was modestly effective in distinguishing endometriosis patients from non-endometriosis patients. miR-451 downregulation in mouse and human oocytes affected pre-implantation embryogenesis by suppressing the Wnt signalling pathway. This miRNA might serve as a novel biomarker of oocyte and embryo quality in assisted reproductive treatment.

## Introduction

Endometriosis is a common oestrogen-related gynaecological disorder that is known to cause serious pelvic pain and infertility and affects 6–10% of reproductive-aged women and 20–50% of infertile women [1, 2]. Previous studies have reported that the quantity and quality of retrieved oocytes are decreased in women with endometriosis, resulting in decreased fertilization rates and poor early embryogenesis during in vitro fertilization (IVF) and embryo transfer [3, 4].

MicroRNAs (miRNAs) are highly conserved, single-stranded, non-coding RNA molecules comprising 20–24 nucleotides. They regulate gene expression, primarily at the posttranscriptional level, through various mechanisms, with positive or negative effects [5, 6]. Several studies have identified the presence of miRNAs in the follicular fluid (FF) of humans [7]. The FF provides a very specialized microenvironment and contains various hormones, proteins, metabolites, and regulatory molecules that play critical roles in oocyte quality and maturation. Recent studies have reported that the miRNA expression profiles of human FF can be used to distinguish high-quality embryos from low-quality ones [8] and affect pathways of ovarian function and follicle development [9]. In addition, we previously found that some miRNAs in the follicular fluid were associated with viable blastocyst formation [10].

However, to date, no studies have reported the miRNA expression profile of the FF of women with endometriosis. Understanding the regulation of miRNAs in the FF and identifying their specific targets and functions could offer novel insights into the aetiology of endometriosis and the associations between endometriosis and abnormal oocytes and embryo development.

The aim of this study was to investigate the miRNA expression profile of the FF of women with endometriosis relative to that of women with male factor infertility. We utilized quantitative reverse transcription polymerase chain reaction (qRT-PCR) to identify the differential expression of miRNAs associated with endometriosis. In addition, we investigated the effects and molecular mechanisms of these miRNAs in oocytes and embryonic development potential by injecting the corresponding inhibitor oligonucleotides into human and mouse oocytes.

## Materials and methods

### Patients

This study involved 30 women with endometriosis and 30 women without endometriosis at Shanghai Ji Ai Genetics and IVF Institute, affiliated with Fudan University, from February to December, 2016. Endometriosis was diagnosed via laparoscopic examination, and the extent of endometriosis was assessed in accordance with the American Society of Reproductive Medicine (ASRM) revised classification [11]. There were 22 cases involving stage-III and 8 cases involving stage IV. (All patients were diagnosed with ovarian endometriomas via pathological biopsy.) Women in the control group all underwent surgery for laparoscopic tubal sterilization, and the absence of endometriosis was confirmed after surgical examination of the abdominal cavity. Women reporting repeatedly high baseline levels of serum follicle stimulating hormone (FSH) (> 15–20 IU/l) or having a severely deformed uterus or any other active infections were excluded.

### Ethical approval

All participants provided written informed consent for the use of FF and discarded metaphase-I (MI) oocytes obtained during the in vitro fertilization (IVF) process. The Institutional Review Committee of Fudan University approved all the protocols (including animal use) of the study.

### Ovarian stimulation and oocyte collection

#### Human oocytes

The study population included 46 patients enrolled in the assisted reproduction programme at the Shanghai Ji Ai Genetics and IVF Institute, affiliated with Fudan University. Eighty-two MI oocytes were obtained from 46 consenting couples. The patients were stimulated with GnRH agonists (Ferring Pharmaceuticals, Switzerland) and recombinant FSH (Gonal F, Merck-Serono, Geneva, Switzerland). Human chorionic gonadotropin (hCG) (Profasi, Merck-Serono) was injected when at least one 18-mm follicle was detected. Ultrasonography-directed oocyte retrieval was performed 36 h after hCG administration. After 2–4 h of incubation, the cumulus masses of the oocytes were removed with a sharp needle and treated with 0.1% hyaluronidase in Dulbecco’s phosphate-buffered saline (DPBS) (w/v) (IrvineScientific, Santa Ana, CA, USA) in preparation for intracytoplasmic sperm injection (ICSI). Only MI oocytes without the first polar body (PB) were used in this study. MI oocytes were cultured in a fertilization medium (Vitrolife) supplemented with 10% HSA in an incubator at 37 °C in 5% CO_2_ in air for 5–7 h until they became MII oocytes [4]. Oocytes were then fertilized using ICSI and incubated in the fertilization medium. Normal fertilization was verified by monitoring the presence of two pronuclei and the second PB at 16–18 h after insemination.

#### Mouse oocytes

Female B6D2F1 mice (6 weeks of age) were superovulated using 5 IU of pregnant mare serum gonadotropin (Ningbo Second Hormone Factory, Ningbo, China) followed by 5 IU of hCG after 48 h (Ningbo Second Hormone Factory). MII oocytes were collected from the oviduct ampullae 13–15 h post-hCG injection. The cumulus-oocyte complexes were introduced into a drop of M2 medium after a 2-min digestion in 300 IU·ml^− 1^ hyaluronidase (Sigma-Aldrich, St. Louis, MO, USA). Denuded oocytes were washed in M2 medium and incubated at 37 °C in 5% CO_2_ and 95% humidified air until injection.

### Sample preparation of FF

The FF samples were collected independently by transvaginal ultrasound-guided puncture and aspiration of follicles with diameters > 18 mm. FF from the first aspirated follicle of each patient was collected carefully and centrifuged at 1300×*g* for 10 min [7]. The supernatant was collected and centrifuged again to completely remove the cellular fragments and blood. The supernatants were then stored at − 80 °C.

### RNA isolation

RNA was extracted using a method proposed in an earlier study [7]. The miRNeasy Kit (QIAGEN, Hilden, Germany) was used for isolation and purification of miRNAs according to the manufacturer’s protocol. Briefly, 500 μl of FF supernatant from each patient was transferred to an Axygen™ centrifuge tube (Corning, Tewksbury, MA, USA) and mixed thoroughly with 700 μl of QIAzol Lysis Reagent (QIAGEN). After 5 min of incubation at 24 °C, 140 μl of chloroform was added to the mixture, and the mixture was vortexed vigorously. The RNA pellet was collected by centrifugation at 3865×*g* for 30 min at 4 °C. The aqueous phase was transferred carefully to a new tube, and a 1.5 volume of absolute ethyl alcohol was added. The RNA pellet was then placed in an RNA-binding column and washed twice. Finally, the pellet was dissolved in 30 μl of nuclease-free H_2_O.

### miRNA analysis and profiling

Thirty nanograms of RNA was initially reverse-transcribed using the Megaplex RT Primers Pools A and B and then pre-amplified with Megaplex Pre-amp Primers Pools A and B. Next, 900 μl of the pre-amplified product was loaded on a TaqMan Array Human MicroRNA Card and run on a Applied Biosystems 7900HT thermocycler in accordance with the manufacturer’s recommended protocol. The cards contained assays for 766 mature miRNAs present in Sanger miRBase version 18.0. MiRNA profiling was performed with the TaqMan Array Human MicroRNA Cards A and B v3.0 (Applied Biosystems) in accordance with the manufacturer’s protocol. The analysis was performed in accordance with a previous study [7]. Detailed data analysis was performed using the Real-Time Statminer software package (Applied Biosystems).

### miRNA validation

To validate the miRNA arrays, we measured the expression levels of the candidate miRNAs by qRT-PCR with TaqMan miRNA assay in each follicular fluid sample in the two groups (30 samples from the endometriosis group and 30 samples from the control group). The expression levels were then normalized based on an internal reference: U6 snRNA [12, 13]. The relative expression levels were calculated as 2^−ΔCt^, where ΔCT = Raw Ct (miRNA)-Raw Ct(U6).

### Microinjection and culture of oocytes

#### Human oocytes

The miR-451 inhibitor was injected via GMOPSplus medium (Vitrolife) using a Nikon (Narishige, Japan) manipulator with a picoinjector (Femtojet, Eppendorf, Hamburg, Germany). The injection was performed via pneumatic pressure. A total of 10–35 pl of the miR-451 inhibitor (50 μmol·l^− 1^) was injected into the cytoplasm of each of the human MII oocytes that had been matured in vitro from the MI stage. An equal volume of negative control (NC) inhibitor (50 μmol·l^− 1^) was injected into control oocytes. The negative control inhibitor was provided by the manufacturer and comprised universal oligonucleotides not homologous to any known mammalian genes. Inhibitor oligonucleotides were synthesized by GenePharma (Shanghai, China). Approximately 10 oocytes were injected each time, and each injection experiment was repeated at least thrice. After injection, the oocytes were introduced into the fertilization medium for 8 h and used for ICSI. Subsequently, embryo development was evaluated at the 8–10-cell and blastocyst stages.

#### Mouse oocytes

A total of 4–10 pl of the miR-451 inhibitor (50 μmol·l^− 1^) was injected into the cytoplasm of mouse MII oocytes. An equal volume of NC inhibitor was injected into the control oocytes. Approximately 60 oocytes were injected each time, and each injection experiment was repeated at least thrice. After injection, the oocytes were introduced into M2 medium for 8 h and then used for IVF. The oocytes injected with miR-451 inhibitor or NC inhibitor were placed in 500 μl EmbryoMax Human Tubal Fluid (Millipore, Billerica, MA, USA) medium under mineral oil. After preincubation of fresh sperm, 100 μl of the sperm suspension (final concentration: 10,000–20,000 spermatozoa·ml^− 1^) was added to the drop containing oocytes. The fertilization dishes were incubated at 37 °C in 5% CO_2_ and 95% humidified air for at least 5 h. The inseminated oocytes were then cultured in EmbryoMaxKSOM (Millipore) medium. The 2-cell formation rate and blastocyst rate were recorded at days 2 and 4 post-fertilization.

### Expression levels of WNT signalling pathway genes in miR-451 inhibitor-injected and control groups

We collected the human and mouse oocytes 8 h (just before insemination) after injection with the miR-451 inhibitor (human oocytes: *n* = 21; mouse oocytes: *n* = 160) or the NC inhibitor (human oocytes: *n* = 20; mouse oocytes: *n* = 95). The miRNeasy Micro Kit (QIAGEN) was used for isolation and purification of RNA from oocytes according to the manufacturer’s protocol [14]. The expression levels of 12 target genes (*WNT4*, *AXIN1*, *COX2*, *CDX2*, *CTNNB1*, *WNT5A*, *WNT3*, *WNT8B*, *CCND1*, c-*MYC*, *ATP2*, and *MMP9*) within or regulating the WNT signalling pathway were measured by qRT-PCR in human/mouse oocytes and in 2-cell and blastocyst-stage embryos. The measurements were then compared between the miR-451 inhibitor-injected and control groups. Prior to PCR, whole transcriptome amplification (TaKaRa, Dalian, China) was performed because the quantity of RNA was limited due to the small number of oocytes. qRT-PCR reactions were performed in triplicate for each sample.

### Statistical analysis

Data are presented as the mean ± SEM or mean ± SD. Student’s t-test was used to assess differences. All statistical analyses were performed using SPSS (version 19.0; SPSS Inc., Chicago, IL, USA). *P* values < 0.05 were considered statistically significant.

## Results

### Clinical and medical characteristics of study participants

**Fig. 1:**
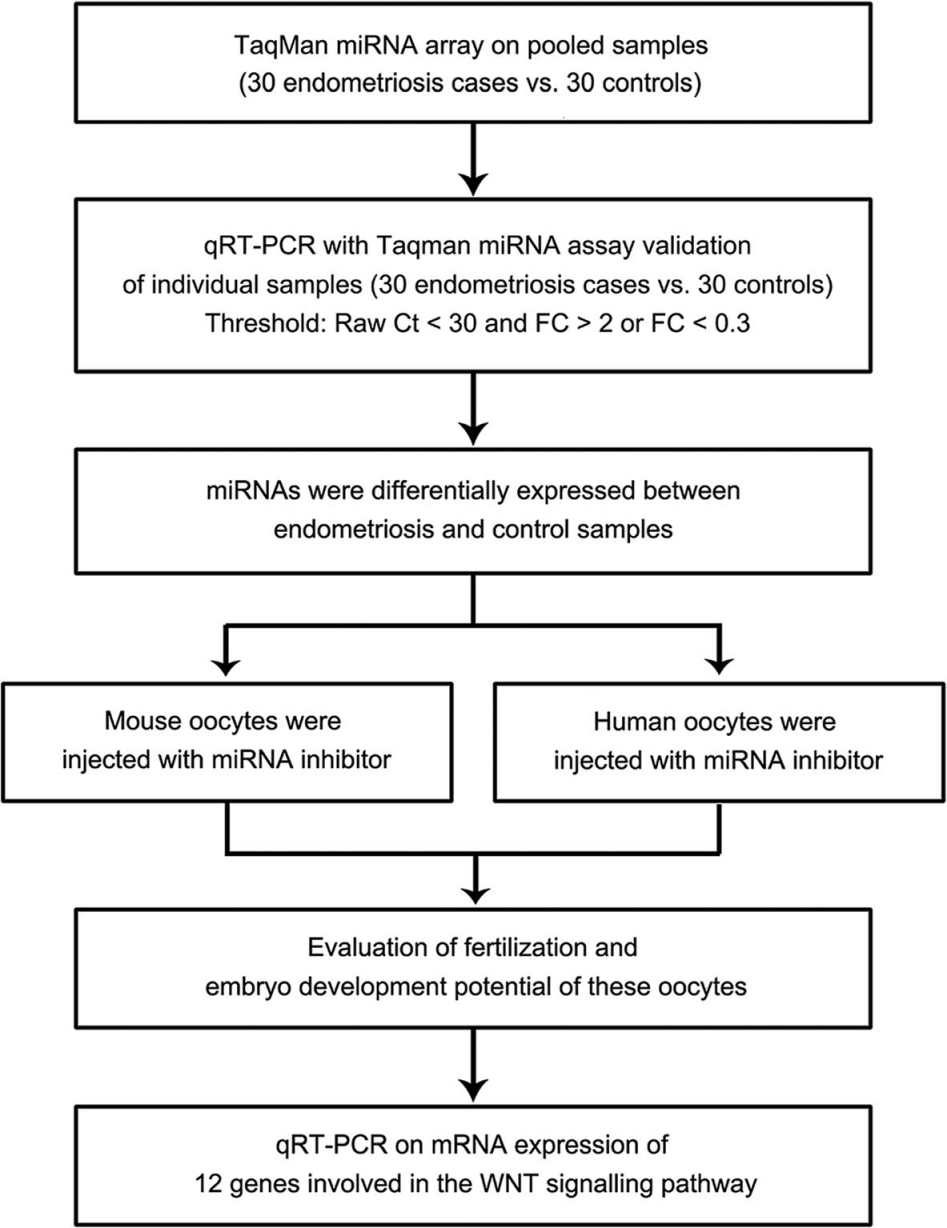
Flow chart of the experimental design

The flow diagram of the study design is shown in Fig. 1. The baseline characteristics of participants with and without endometriosis are listed in Table 1. The two groups did not differ in age, years of infertility, body mass index (BMI), cycle length, endometrial thickness, proportion of IVF/ICSI, or hormone levels. However, the number of oocytes, fertilization rate, and number of available embryos were significantly greater in the controls than in the endometriosis patients (all *P* < 0.01, Table 1).

**Table 1:** Clinical characteristic of infertile patients with endometriosis and controls

	Endometriosis (*n* = 139)	Controls (*n* = 184)	*P*-values
Maternal age (years)	33.2 ± 5.7	32.6 ± 5.4	NS
BMI (kg/m^2^)	22.1 ± 2.8	21.7 ± 2.9	NS
Years of infertility	4.7 ± 1.9	4.5 ± 1.8	NS
Cycle length (days)	31.4 ± 5.4	32.5 ± 8.9	NS
Endometrial thickness (mm)	10.63 ± 2.24	10.56 ± 2.20	NS
Proportion of IVF (%)	86 (61.9)	110 (59.8)	NS
Proportion of ICSI (%)	53 (38.1)	74 (40.2)	NS
Day-3 E2 (pg/ml)	35.8 ± 16.3	36.6 ± 21.0	NS
Day-3 P4 (ng/ml)	0.8 ± 1.0	0.7 ± 0.4	NS
Day-3 LH (mIU /ml)	5.5 ± 4.1	4.6 ± 3.7	NS
Day-3 FSH (mIU/ml)	8.8 ± 3.3	7.5 ± 2.2	NS
Normal fertilization rate (%)	65.1 ± 25.6	74.6 ± 22.3	< 0.01
Number of oocytes	7.3 ± 4.3	10.7 ± 4.7	< 0.01
Number of available embryos	4.2 ± 2.9	6.7 ± 2.9	< 0.01

The controls were normal responders without endometriosis, who were undergoing treatment for male factor infertility. *NS* no significant difference. *P*-values: Unpaired *t*-test. *BMI* body mass index, *IVF* in vitro fertilization, *ICSI* intracytoplasmic sperm injection, *E2* oestradiol, *P4* progesterone, *LH* luteinizing hormone, *FSH* follicle stimulating hormone

### FF miRNA profiles and the identification of differentially expressed miRNAs in participants with and without endometriosis

**Fig. 2:**
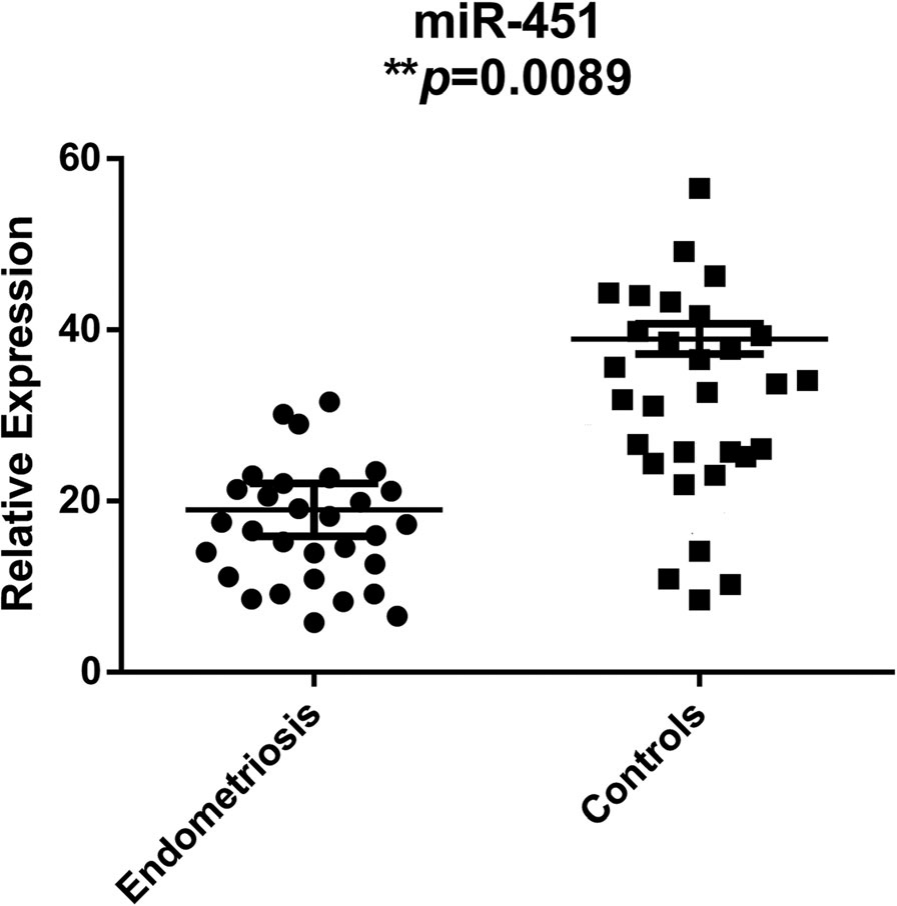
The relative expression levels of hsa-miR-451 in the endometriosis group and control group. Scatter plots depicting the relative expression levels of hsa-miR-451 in the two groups are shown. Differences were evaluated via unpaired *t*-test. *P < 0.05; **P < 0.01. Mean ± SD shown by bars

The finding that embryo quality was higher in the control group than in the endometriosis group suggested that miRNAs with expression level differences between the endometriosis group and controls might play a role in embryo development potential. MiRNAs with Raw Ct < 30 have been considered to be highly expressed in many studies [10, 15]. Thus, to identify and verify differentially expressed miRNAs associated with embryo development potential, we chose candidate miRNAs in endometriosis group with Raw Ct (miRNA) < 30, thereby excluding those miRNAs with low expression levels. MiRNAs with high expression levels (Raw Ct < 30) are listed in Additional file 1: Table S1. As shown in Table 2, in the endometriosis group relative to the control group, 11 miRNAs (miR-1260, miR-145, miR-125a, miR-21, miR-628, miR-542, miR-223, miR-663, miR-378, miR-23a and miR-451) were downregulated, and 7 miRNAs (miR-766, miR-133, miR-191, miR-720, miR-143, miR-29c and miR-203) were upregulated. These miRNAs were chosen for subsequent verification analysis. These miRNAs had the highest relative expression quantities, and the differences between the endometriosis group and controls were based on the miRNA profiling results. We measured the expression levels of these candidate miRNAs by qRT-PCR with TaqMan® miRNA assay in each follicular fluid sample of the two groups. As indicated in Fig. 2, among the 18 candidate miRNAs, miR-451 had an expression level that was significantly lower in the endometriosis group than in the control group (*P* = 0.0089) (Fig. 2). The expression levels of the remaining miRNAs were not significantly different between the two groups (Additional file 2: Figure S1).

**Table 2:** MicroRNAs with high expression levels, identified by miRNA array between follicular fluid samples from control and endometriosis patients

MicroRNAs	Endometriosis			Controls			
	Raw Ct	ΔCt	RQ	Raw Ct	ΔCt	RQ	FC
hsa-miR-766	25.3422	7.1551	7.0162	29.3191	10.3442	0.7693	9.1204
hsa-miR-133	23.2389	5.0518	30.1479	25.9875	7.0126	7.7446	3.8928
hsa-miR-191	25.9964	7.8093	4.4583	28.9539	9.9790	0.9909	4.4993
hsa-miR-720	26.9596	8.7725	2.2867	29.9853	11.0104	0.4848	4.7171
hsa-miR-143	25.3576	7.1705	6.9417	29.9635	9.9886	0.9843	7.0523
hsa-miR-29c	24.1276	5.9405	16.2829	27.5496	8.5747	2.6228	6.2083
hsa-miR-203	21.2276	3.0405	121.5397	22.9652	5.9903	15.7304	7.7264
hsa-miR-1260	22.9617	4.7746	36.5344	21.9646	2.9897	125.8956	0.2902
hsa-miR-145	25.8444	7.6573	4.9536	24.7811	5.8062	17.8714	0.2772
hsa-miR-125a	28.3772	10.1901	0.8560	25.8664	7.8915	4.2114	0.2033
hsa-miR-21	24.7855	6.5984	10.3201	23.9475	3.9726	63.6984	0.1620
hsa-miR-628	22.9150	4.7279	37.7364	21.8117	2.8368	139.9710	0.2696
hsa-miR-542	25.9240	7.7369	4.6877	22.2701	3.2952	101.8699	0.0460
hsa-miR-223	26.1911	8.0040	3.8954	23.9329	4.9580	32.1731	0.1211
hsa-miR-663	27.1383	8.9512	2.0203	25.8087	5.8338	17.5328	0.1152
hsa-miR-378	28.6822	10.4951	0.6929	26.7895	7.8146	4.4419	0.1560
hsa-miR-23a	27.5550	9.3679	1.5135	22.7249	6.7500	9.2907	0.1629
hsa-miR-451	21.3157	3.1286	114.3398	19.0286	0.0537	963.4622	0.1187

Internal reference: snRNA U6. ΔCT = Raw Ct (miRNA)-Raw Ct(U6). Inclusion criteria: Raw Ct < 30. Fold Change (FC) = RQ (Endometriosis)/RQ (Controls), FC > 2 or FC < 0.3, RQ, relative quantity, RQ = 2 ^-ΔCt^ *1000

### Knockdown of mmu-miR-451 in mouse oocytes influences early embryonic potential

**Fig. 3:**
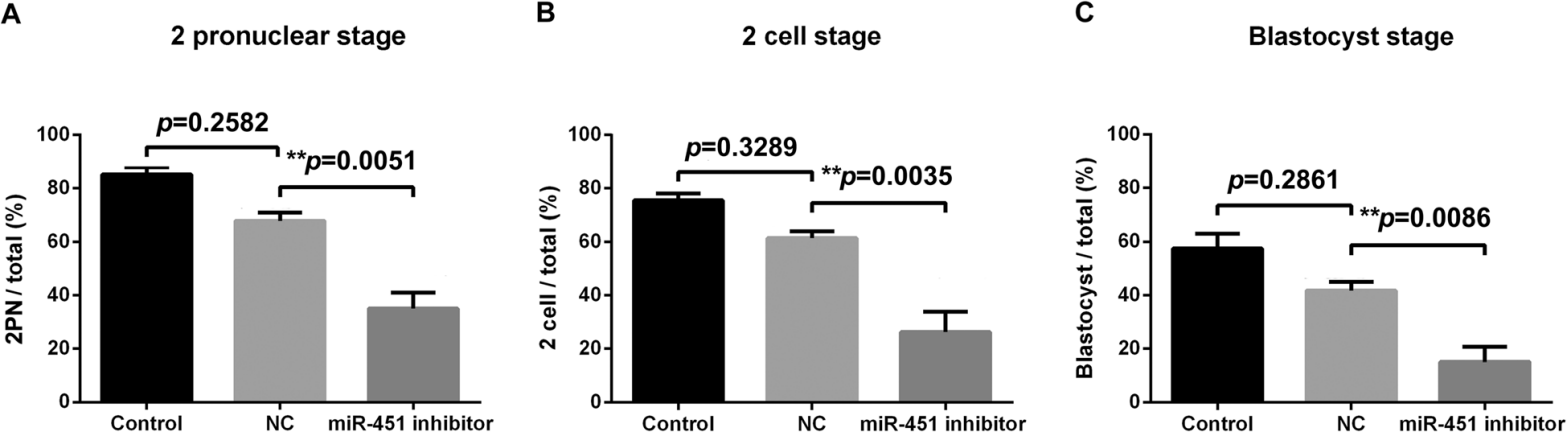
Statistical results of the 2 pronuclear (2PN), 2-cell, and blastocyst stages of mouse oocytes after injection with either miR-451 inhibitor (*n* = 132) or negative control (NC) inhibitor (*n* = 95) and oocytes of the untreated control group (*n* = 245). The proportions of oocytes in the untreated control, NC inhibitor, and miR-451 inhibitor groups that developed into the 2PN stage (**a**) were 86.57% ± 7.15, 69.37% ± 7.48, and 35.12% ± 4.78%, respectively; into the 2-cell stage (**b**) were 78.59% ± 6.05, 61.63% ± 6.51, and 27.38% ± 5.31%, respectively; and into the blastocyst stage (**c**) were 59.43% ± 4.86, 41.26% ± 4.89, and 18.23% ± 2.32%, respectively. All experiments were repeated at least thrice. Unpaired *t*-test, *P < 0.05; **P < 0.01; ***P < 0.001. NC, negative control

As shown in Fig. 3, mouse embryogenesis was markedly affected by miR-451 inhibitor injection. The proportions of oocytes that developed into 2PN, 2-cell, and blastocyst-stage embryos were 35.12% ± 4.78, 27.38% ± 5.31, and 18.23% ± 2.32%, respectively, in the miR-451 inhibitor group (*n* = 132); 69.37% ± 7.48, 61.63% ± 6.51, and 41.26% ± 4.89%, respectively, in the NC group (*n* = 95); and 86.57% ± 7.15, 78.59% ± 6.05, and 59.43% ± 4.86%, respectively, in the non-injected group (*n* = 245).

### Abnormal expression levels of Wnt components in mouse oocytes and embryos in the mmu-miR-451 inhibitor-injected group

**Fig. 4:**
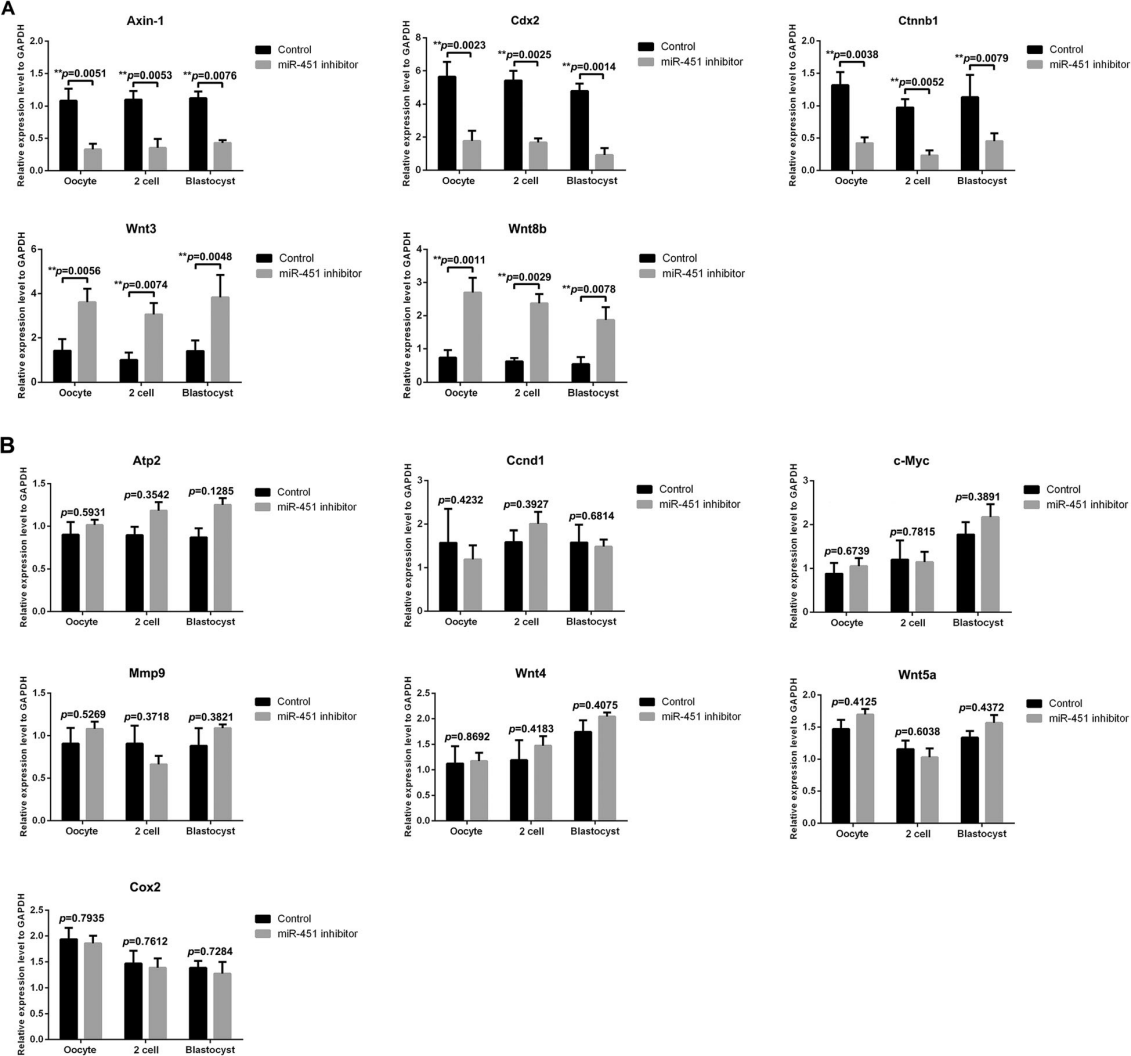
Significant and non-significant expression differences of Wnt components in mouse oocytes and in 2-cell- and blastocyst-stage embryos between the miR-451 inhibitor-injected and control groups. The histograms present five genes that are within or regulate the Wnt signalling pathway with markedly different expression levels (**a**) and seven genes that are within or regulate the Wnt signalling pathway with non-significant differences in expression level (**b**) between the miR-451 inhibitor (*n* = 160) and control (*n* = 95) groups. All experiments were repeated at least thrice. Unpaired *t*-test; *P < 0.05; **P < 0.01; ***P < 0.001

mRNA expression profiles for 12 genes of the Wnt signalling pathway were generated via qRT-PCR in the miR-451 inhibitor and NC inhibitor-injected groups. The results showed that three predicted genes, *Axin1*, *Cdx2*, and *Ctnnb1*, exhibited significantly decreased expression in mouse oocytes and 2-cell- and blastocyst-stage embryos in the miR-451 inhibitor group relative to the controls. The expression of two other genes, *Wnt3* and *Wnt8b*, was significantly increased in the miR-451 inhibitor group relative to the controls (*P* < 0.01, Fig. 4a). The expression of seven other genes (*Ccnd1*, *Wnt4*, *Mmp9*, c-*Myc*, *Cox2*, *Atp2*, and *Wnt5a*) did not significantly differ between these groups (*P* > 0.05, Fig. 4b).

### Knockdown of hsa-miR-451 in human oocytes influences early embryonic development

**Fig. 5:**
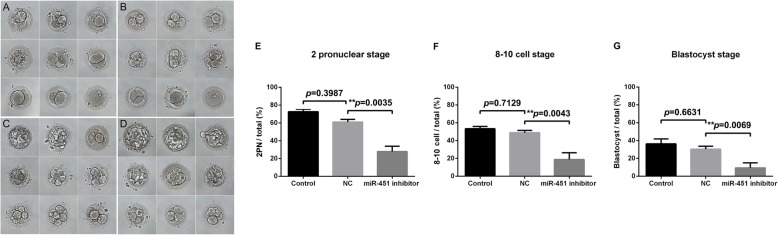
Morphology and statistical results of the 2 pronuclear (2PN), 8–10-cell, and blastocyst stages of human oocytes after injection with either miR-451 inhibitor or negative control (NC) inhibitor and oocytes of the untreated control group. Morphology of the 2PN, 8–10-cell and blastocyst stages of the miR-451 inhibitor-injected (*n* = 22), NC inhibitor-injected (*n* = 20), and untreated (*n* = 19) oocytes. **a** The 8–10-cell stage of abnormal embryos from oocytes microinjected with miR-451 inhibitor; **b** The 8–10-cell stage of normal embryos from oocytes microinjected with NC inhibitor; **c** The blastocyst stage of abnormal embryos from oocytes microinjected with miR-451 inhibitor; **d** The blastocyst stage of normal embryos from oocytes microinjected with NC inhibitor. Scale bar = 50 μm. The proportions of oocytes in the untreated, NC inhibitor, and miR-451 inhibitor groups that developed into the 2PN stage (**e**) were 72.64% ± 5.62, 61.25% ± 5.71, and 28.96% ± 3.29%, respectively; into the 8–10-cell stage (**f**) were 53.42% ± 4.78, 49.15% ± 4.28, and 19.81% ± 3.73%, respectively; and into the blastocyst stage (**g**) were 37.54% ± 3.72, 31.16% ± 4.89, and 9.23% ± 2.32%, respectively. All experiments were repeated at least thrice. Unpaired *t*-test; *P < 0.05; **P < 0.01; ***P < 0.001. NC, negative control

As shown in Fig. 5, human embryo development was significantly affected by miR-451 inhibitor injection. The proportions of oocytes that developed into 2PN, 8–10 cell, and blastocyst-stage embryos were 28.96% ± 3.29, 19.81% ± 3.73, and 9.23% ± 2.32%, respectively, in the miR-451 inhibitor group (*n* = 22); 61.25% ± 5.71, 49.15% ± 4.28, and 31.16% ± 4.89%, respectively, in the NC group (*n* = 20); and 72.64% ± 5.62, 53.42% ± 4.78, and 37.54% ± 3.72%, respectively, in the non-injected group (*n* = 19). Because of the physical damage due to injection, the proportions of 8–10 cell and blastocyst-stage embryos were lower (although not to a significant level) in the injection groups than in the non-injected group, which we considered acceptable.

### Abnormal expression levels of WNT components in human oocytes and embryos in the hsa-miR-451 inhibitor-injected group

**Fig. 6:**
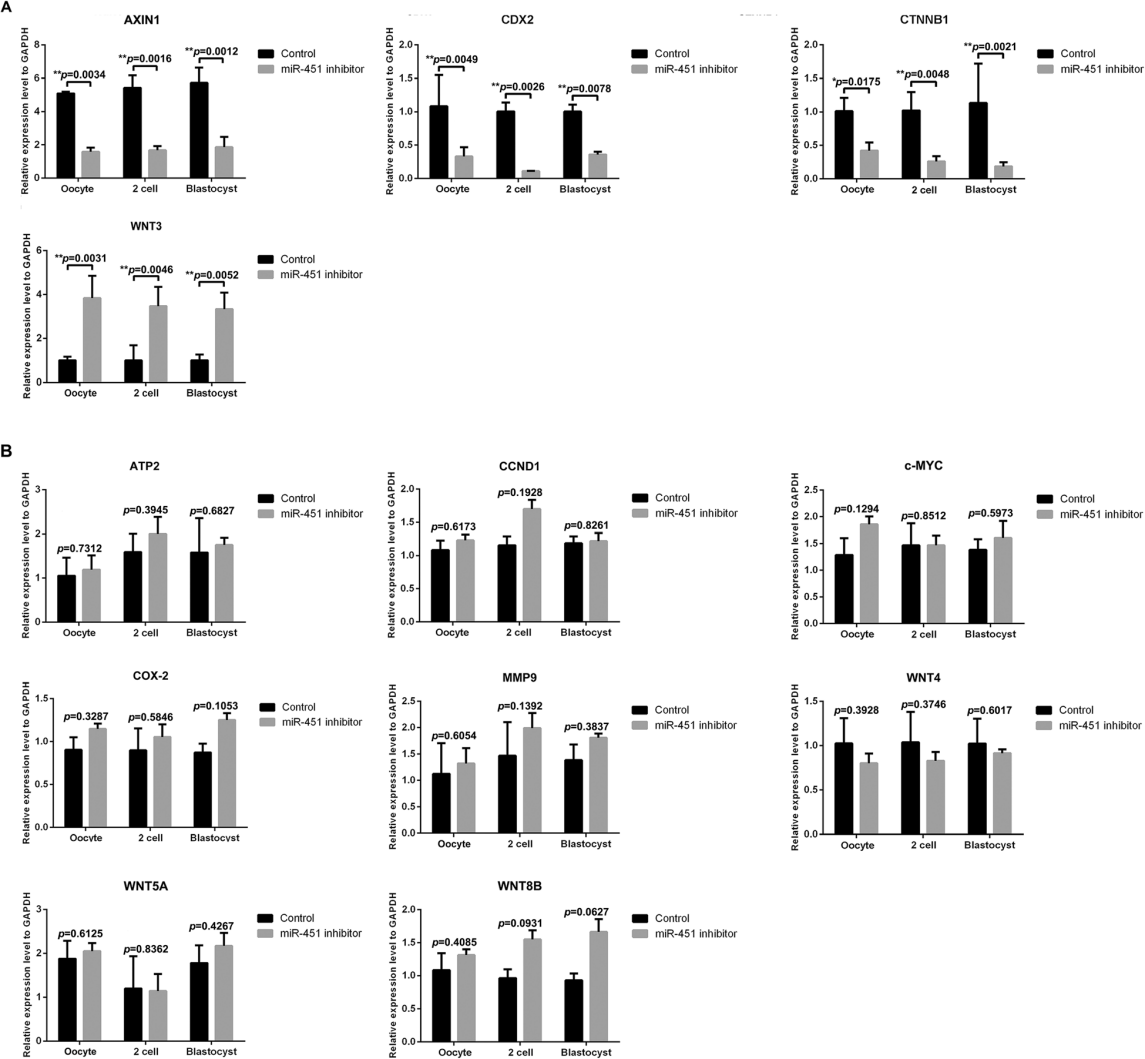
Significant and non-significant expression differences of WNT components in human oocytes and in 2-cell- and blastocyst-stage embryos between the miR-451 inhibitor-injected and control groups. The histograms present four crucially differentially expressed genes that are within or regulate the WNT signalling pathway (**a**) and eight genes that are within or regulate the WNT signalling pathway with non-significant expression differences (**b**) between the miR-451 inhibitor (*n* = 21) and control (*n* = 20) groups. All experiments were repeated at least thrice. Unpaired *t*-test; *P < 0.05; **P < 0.01; ***P < 0.001

Consistent with the results from the mouse samples, the results from the human samples indicated that the three predicted genes, *AXIN1*, *CDX2*, and *CTNNB1*, were markedly downregulated in the miR-451 inhibitor-injected group relative to the control group. Only *WNT3* was substantively upregulated in human oocytes and in 2-cell and blastocyst-stage embryos in the miR-451 inhibitor group relative to the control group (*P* < 0.01, Fig. 6a). The expression of the other eight genes (*CCND1*, *WNT4*, *MMP9*, c-*MYC*, *COX2*, *ATP2*, *WNT5A*, and *WNT8B*) were not found to be significantly different between the miR-451 inhibitor (*n* = 21) and control (*n* = 20) groups (*P* > 0.05, Fig. 6b).

## Discussion

In the present study, the clinical data indicated that the number of oocytes, fertilization rate, and number of available embryos were significantly reduced in endometriosis patients relative to non-endometriosis patients. The TaqMan miRNA arrays and qRT-PCR validated the finding that hsa-miR-451 showed significantly lower expression in the endometriosis group than in the control group. Subsequently, our in vitro study suggested that miR-451 significantly affected embryo development potential. Functional analysis of mouse/human oocytes and embryos showed that the aberrant expression of WNT signalling pathway genes might negatively affect oocyte competence for fertilization and pre-implantation embryo development. To the best of our knowledge, this is the first quantitative assessment of FF miRNAs that could explain aberrant oocyte and embryo development in women with endometriosis.

FF is a complex mixture of proteins, hormones, vitamins, cytokines, and metabolites, and multiple studies have indicated that the constituents of FF may affect the acquisition of oocyte competence in women with endometriosis [16]. The presence of miRNAs in human FF was first described by Sang et al. (2013) [7]. They demonstrated that some miRNAs could regulate steroidogenesis in vitro and showed that their expression levels were correlated with polycystic ovary syndrome (PCOS) in vivo. Recently, it was reported that miRNA levels in human FF may lead to downstream events that could potentially determine fertilization and day-3 embryo morphology [8]. In addition, it was shown that miRNA expression profiling in FF samples could provide biomarkers to identify women with PCOS and to predict blastocyst formation and clinical pregnancy outcome [17]. However, there have been no studies on the relationships between miRNAs in the FF of endometriosis patients and oocyte quality, fertilization, and early embryo development. In the current study, we hypothesized that miRNAs in the FF of endometriosis patients are associated with oocyte and pre-implantation embryo development.

In this study, we observed significantly lower expression of miR-451 in the FF in women with endometriosis than in healthy women. This finding is consistent with previous reports that miR-451 expression was greatly decreased in eutopic and ectopic endometria in baboons and women with endometriosis [18]. To assess the role of miR-451 in early embryonic development, miR-451 inhibitor oligonucleotides were used to suppress it in mouse/human oocytes. This study demonstrated that the fertilization, cleavage, and blastocyst rate of mouse/human oocytes were strongly influenced by the knockdown of miR-451, which further suggested that miR-451 has a significant impact on embryo development potential. Our conclusion differs from that of previous research [19] in which loss-of-function mediated by a lentiviral miR-451 sponge vector (LV-miR-451 sponge) or miR-451 inhibitor reduced the number of embryo implantations but had little effect on fertilization [19]. However, the previous study differed significantly from ours in the experimental methods used. To efficiently decrease miR-451 expression level, we performed microinjection of the miR-451 inhibitor into the cytoplasm of mouse/human oocytes, whereas Li et al. injected the cornu uteri of an experimental mouse model with LV-miR-451 sponge or the negative control vector.

Although some studies have suggested that miRNA function is repressed in oocytes [20, 21], others have reported an association between altered miRNA expression levels and both oocyte development and early embryogenesis [8, 9, 22, 23]. miRNAs play key roles in numerous signalling pathways, and there is evidence for roles of miR-451 in the regulation of multiple signalling pathways, including the Wnt signalling, AMPK signalling, and IL-6R-STAT3 pathways [24, 25]. Furthermore, several studies have reported that the components of the Wnt signalling pathway are involved in ovarian folliculogenesis [26] and pre-implantation embryogenesis, including fertilization [27], embryonic development [28], trophectoderm specification in human blastocysts [29], promotion of blastocyst hatching [30], and embryo implantation [31]. In addition, it has been suggested that the Wnt signalling pathway is regulated by miR-451 [32]. These observations indicate that the Wnt signalling pathway plays a role in early pre-implantation embryogenesis, and we postulate that this may be altered upon miR-451 downregulation.

Therefore, we analysed the expression of 12 genes associated with the Wnt signalling pathway in treated mouse oocytes. *Axin1*, *Cdx2*, *Ctnnb1*, *Wnt3*, and *Wnt8b* were aberrantly expressed in the miR-451 inhibitor-treated group, indicating the suppression of Wnt signalling pathway. Among the five differentially expressed genes, *Axin1*, *Cdx2*, *Ctnnb1*, and *Wnt3* are crucial in oocyte maturation, fertilization, and early embryogenesis [29, 33–35].

Previous evidence suggests that Cdx2 is important for proper pre-implantation embryogenesis, owing to its role in the specification of the trophectoderm lineage from the morula stage [35]. Knockdown of maternal and zygotic Cdx2 was found to significantly reduce the developmental potential of mouse oocytes and increase cell death from the morula stage onward [35]. These results suggest that Cdx2 downregulation after the micro-injection of oocytes with miR-451 inhibitor may reduce the developmental potential of mouse oocytes to the blastocyst stage, thus increasing cell death and inhibiting the specification of the trophectoderm lineage. Similar to Cdx2, Ctnnb1 was downregulated in the miR-451 inhibitor-treated group. The inhibition of Ctnnb1 from the zygote stage has been found to reduce blastocyst diameter and number and partially increase embryo fragmentation [33, 36]. In the present study, the downregulation of Ctnnb1 after oocyte injection with miR-451 inhibitor led to decreased cell number in the embryo and increased embryo fragmentation. Furthermore, this downregulation might have affected the day 3 embryo cell number and fragmentation in patients receiving ICSI, whose FF had relatively low levels of miR-451.

The present investigation has several limitations. First, all the patients included had stage III/IV endometriosis; thus, further validation of our results in patients with early stages of the disease is necessary. Second, the human MII oocytes used in this study had been matured in vitro from the MI stage. The fact that these oocytes were exposed to gonadotrophic stimulation but failed to mature in vivo might reflect their low quality; this should be considered when drawing conclusions from the data.

## Conclusions

In conclusion, this study is the first to suggest that the differential expression of miRNAs in human FF may help explain aberrant oocyte and embryo development in women with endometriosis. The downregulation of miR-451 in mouse and human oocytes negatively affected pre-implantation embryo development by suppressing the expression of the WNT signalling pathway. In addition, the edification of miRNAs in the FF of women with endometriosis may improve the quality of diagnosis and therapy for endometriosis; these miRNAs could serve as novel biomarkers of oocyte and embryo quality in assisted reproductive treatment.

## Supplementary information

**Additional file 1: Table S1.** MicroRNAs with high expression levels (Raw Ct < 30), identified by miRNA array between follicular fluid samples from control and endometriosis patients.

**Additional file 2: Figure S1.** The relative expression levels of 17 miRNAs in the endometriosis group and control group. Scatter plots present ten miRNAs that were downregulated with non-significant differences in relative expression levels (A) and seven miRNAs that were upregulated with non-significant differences in relative expression levels (B) between the endometriosis group and control group. Unpaired *t*-test, *P < 0.05; **P < 0.01. Mean ± SD shown by bars.

## Data Availability

All data generated or analysed during this study are included in this published article and its supplementary information files.

## Abbreviations

FF: The follicular fluid
ICSI: Intracytoplasmic sperm injection
IVF: In vitro fertilization
PB: The first polar body
qRT-PCR: quantitative reverse transcription polymerase chain reaction
