# Supplementary material for: MicroRNA-451 is downregulated in the follicular fluid of women with endometriosis and influences mouse and human embryonic potential
Source: Reprod Biol Endocrinol. 2019 Nov 19;17:96. doi: 10.1186/s12958-019-0538-z (PMC6862852; doi:10.1186/s12958-019-0538-z)
Supplement: Supplementary file 1 — Additional file 1: Table S1. MicroRNAs with high expression levels (Raw Ct < 30), identified by miRNA array between follicular fluid samples from control and endometriosis patients. [file 12958_2019_538_MOESM1_ESM.docx]

**Table S1 MicroRNAs with high expression levels (Raw Ct < 30), identified by miRNA array between follicular fluid samples from control and endometriosis patients**

| **MicroRNAs** | **Raw Ct** | |
| --- | --- | --- |
|  | **Endometriosis** | **Controls** |
| hsa-miR-766 | 25.3422 | 29.3191 |
| hsa-miR-133 | 23.2389 | 25.9875 |
| hsa-miR-191 | 25.9964 | 28.9539 |
| hsa-miR-720 | 26.9596 | 29.9853 |
| hsa-miR-143 | 25.3576 | 28.9635 |
| hsa-miR-29c | 24.1276 | 27.5496 |
| hsa-miR-203 | 21.2276 | 24.9652 |
| hsa-miR-1260 | 22.9617 | 21.9646 |
| hsa-miR-145 | 25.8444 | 24.7811 |
| hsa-miR-125a | 28.3772 | 26.8664 |
| hsa-miR-21 | 24.7855 | 22.9475 |
| hsa-miR-628 | 22.9150 | 21.8117 |
| hsa-miR-542 | 25.9240 | 22.2701 |
| hsa-miR-223 | 26.1911 | 23.9329 |
| hsa-miR-663 | 27.1383 | 24.8087 |
| hsa-miR-378 | 28.6822 | 26.7895 |
| hsa-miR-23a | 27.5550 | 25.7249 |
| hsa-miR-451 | 21.3157 | 19.0286 |
| hsa-miR-15b | 28.9734 | 28.5290 |
| hsa-miR-25 | 26.5382 | 27.9273 |
| hsa-miR-30c | 23.9329 | 24.5202 |
| hsa-miR-31 | 26.7054 | 26.9741 |
| hsa-miR-99a | 26.9897 | 27.2042 |
| hsa-miR-103 | 26.7671 | 27.8958 |
| hsa-miR-324-3p | 27.0592 | 27.9886 |
| hsa-miR-375 | 27.2833 | 25.9718 |
| hsa-miR-382 | 23.5571 | 24.1127 |
| hsa-miR-148a | 27.1318 | 26.9435 |
| hsa-miR-181a | 28.9539 | 29.4582 |
| hsa-miR-192 | 28.9652 | 28.8419 |
| hsa-miR-200a | 26.1044 | 26.0125 |
| hsa-miR-532 | 27.7736 | 29.1053 |
| hsa-miR-590-5p | 27.6197 | 27.9597 |
| hsa-miR-660 | 25.1280 | 24.9076 |
| hsa-miR-874 | 25.8026 | 26.9559 |
| hsa-miR-212 | 24.1410 | 25.4931 |
| hsa-miR-376c | 24.1393 | 25.0377 |
| hsa-miR-520e | 27.7280 | 29.0386 |
| hsa-miR-130a | 26.1911 | 26.8558 |
| hsa-miR-139-5p | 26.5550 | 27.9544 |
| hsa-miR-210 | 21.4100 | 22.0481 |
| hsa-miR-16 | 21.8176 | 21.9545 |
| hsa-miR-24 | 21.5286 | 21.9425 |
| hsa-miR-425-5p | 25.8999 | 24.3666 |
| hsa-miR-509-5p | 27.7748 | 27.9656 |
