# Supplementary figures and images for: MicroRNA-451 is downregulated in the follicular fluid of women with endometriosis and influences mouse and human embryonic potential
Source: Reprod Biol Endocrinol. 2019 Nov 19;17:96. doi: 10.1186/s12958-019-0538-z (PMC6862852; doi:10.1186/s12958-019-0538-z)

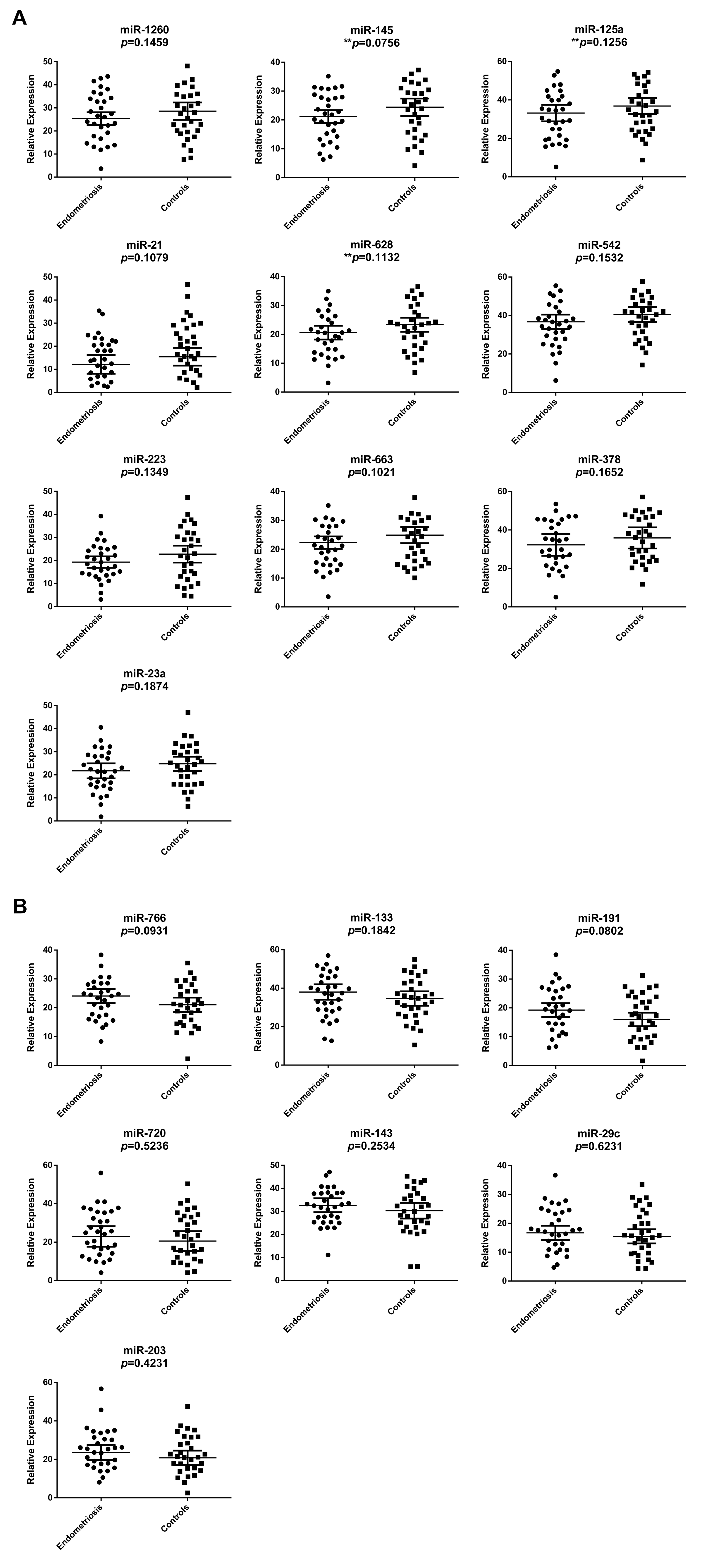

Supplement: Supplementary file 2 — Additional file 2: Figure S1. The relative expression levels of 17 miRNAs in the endometriosis group and control group. Scatter plots present ten miRNAs that were downregulated with non-significant differences in relative expression levels (A) and seven miRNAs that were upregulated with non-significant differences in relative expression levels (B) between the endometriosis group and control group. Unpaired t-test, *P < 0.05; **P < 0.01. Mean ± SD shown by bars. [file 12958_2019_538_MOESM2_ESM.tif]
